# Elevated Bmi-1 expression is associated with dysplastic cell transformation during oral carcinogenesis and is required for cancer cell replication and survival

**DOI:** 10.1038/sj.bjc.6603529

**Published:** 2006-12-19

**Authors:** M K Kang, R H Kim, S J Kim, F K Yip, K-H Shin, G P Dimri, R Christensen, T Han, N-H Park

**Affiliations:** 1 UCLA School of Dentistry, Los Angeles, CA 90095, USA; 2 UCLA Jonsson Comprehensive Cancer Center, Los Angeles, CA 90095, USA; 3 Evanston Northwestern Healthcare Research Institute, Feinberg School of Medicine, Northwestern University, Evanston, IL 60201, USA; 4 David Geffen School of Medicine at UCLA, Los Angeles, CA 90095, USA

**Keywords:** oral cancer, polycomb, Bmi-1, keratinocytes, p16^INK4A^, RNA interference

## Abstract

Bmi-1 is a polycomb group protein that was identified as c-myc cooperating oncogene in murine lymphomagenesis. The current study was undertaken to determine the role of Bmi-1 in human oral carcinogenesis. Bmi-1 protein and RNA expression levels were markedly enhanced in the cells of oral squamous cell carcinomas (OSCC) compared with that of normal human oral keratinocytes (NHOK). Enhanced-Bmi-1 expression was also detected *in situ* in the archived oral mucosal tissues with cancerous and precancerous histopathology, including that of mild epithelial dysplasia. Thus, Bmi-1 expression occurs at a very early stage in oral carcinogenesis. To determine the biological role of Bmi-1 in cell proliferation, endogenous Bmi-1 was knocked down in actively proliferating SCC4 cells and NHOK by RNA interference. After Bmi-1 knockdown, cell replication was severely retarded. However, the expression of p16^INK4A^, a known cellular target of Bmi-1, was not changed in cells with or without Bmi-1 knockdown. Furthermore, Bmi-1 knockdown in HOK-16B-BaP-T cells, in which the p16^INK4A^/pRb pathway was abrogated, led to immediate arrest of replication and loss of viable cells. Thus, our data suggest that Bmi-1 may act through p16^INK4A^-independent pathways to regulate cellular proliferation during oral cancer progression.

Bmi-1 is a polycomb group (PcG) transcription repressor originally identified as a c-myc cooperating oncogene in murine lymphomas (van Lohuizen *et al*, 1991; Pirrotta, 1998). Proliferative capacity of leukaemic and normal haemopoietic stem cells derived from *Bmi-1*^*-/-*^ mice was compromised, suggesting the possible role of Bmi-1 in the maintenance of tumour stem cell phenotype (Lessard and Sauvageau, 2003). Bmi-1 is one of the core subunits of the polycomb repressive complex 1, which regulates the diverse biological processes, including X chromosome inactivation, carcinogenesis, and stem cell renewal (Li *et al*, 2006; Sparmann and van Lohuizen, 2006). Cellular target genes of Bmi-1 have been identified and include *ink4a* and *ink4b* loci, encoding p16^INK4A^, p19^ARF^, and p15^INK4B^ (Jacobs *et al*, 1999). Bmi-1 is believed to promote cellular proliferation by repressing the expression of the *ink4a* locus, which plays pivotal roles in the onset of cellular senescence in many different types of human somatic cells (reviewed in Itahana *et al*, 2004). During normal replication of primary human cells, Bmi-1 expression level is decreased notably in senescent cells (Itahana *et al*, 2003). When overexpressed, Bmi-1 is able to extend the replicative life span of human diploid fibroblasts (Itahana *et al*, 2003) and immortalise the post-selection population of human mammary epithelial cells by activation of endogenous telomerase gene, *hTERT* (Dimri *et al*, 2002). Therefore, in normal human cells, downregulation of Bmi-1 expression may be important for the establishment of the senescence program.

Cellular senescence is physiologically important because it is a potent tumor suppressor mechanism that must be overcome for cells to be immortalised and transformed (Campisi, 2005; Dimri, 2005). The molecular mechanisms of senescence have therefore been investigated extensively and yielded several important regulators of senescence (Itahana *et al*, 2004). The intrinsic, telomere-dependent senescence is triggered by accumulation of shortened and dysfunctional telomeres with altered telomeric state (Harley *et al*, 1990; Karlseder *et al*, 2002). The extrinsic senescence is telomere-independent and triggered in cells after exposure to environmental factors, such as genotoxic stress, *in vitro* culture shock, and oncogenic stimuli (Serrano *et al*, 1997; Ramirez *et al*, 2001; Itahana *et al*, 2004; Chen *et al*, 2005; Dimri, 2005). The onset of extrinsic senescence is frequently associated with induction of p16^INK4A^ (Itahana *et al*, 2004). The mechanisms resulting in p16^INK4A^ upregulation during senescence are not fully understood, although recent studies found a positive control by *ETS1* and negative regulation of its expression by *Id-1*, *Bmi-1*, and *CBX7* (Ohtani *et al*, 2001; Itahana *et al*, 2003; Gil *et al*, 2004). In particular, Bmi-1 overexpression is detected in several types of human cancers (Bea *et al*, 2001; Kim *et al*, 2004a, 2004b; Breuer *et al*, 2005), leading to the hypothesis that Bmi-1 facilitates tumorigenesis by nullifying the p16^INK4A^-mediated extrinsic senescence pathway.

The current study was undertaken to investigate the role of Bmi-1 in oral carcinogenesis and its functional relationship with p16^INK4A^. We report enhanced expression of Bmi-1 in preneoplastic and cancerous oral epithelial tissues compared with the normal counterparts. The cell lines established from oral squamous cell carcinomas (OSCC) also expressed notably higher level of Bmi-1 compared with those of normal human oral keratinocytes (NHOK). However, the expression levels of Bmi-1 and p16^INK4A^ were poorly correlated in the OSCC cells. Knockdown of endogenous Bmi-1 led to rapid inhibition of cell proliferation in the OSCC cells, regardless of the presence of functional p16^INK4A^/pRb cell cycle inhibitory pathway in these cells. Thus, our results indicate that Bmi-1 may act through p16^INK4A^-independent pathways to regulate cellular proliferation during oral carcinogenesis.

## MATERIALS AND METHODS

### Cells and cell culture

Primary NHOK cultures were prepared from separated keratinising oral epithelial tissue discarded during routine oral surgery procedures. The cells were cultured in keratinocyte growth medium (KGM) containing a low level (0.15 mM) of Ca^++^ and supplementary growth factor bullet kit (Cambrex, East Rutherford, NJ, USA). The detailed method of primary culture establishment can be found elsewhere (Kang *et al*, 2000). BaP-T, SCC4, SCC9, SCC15, FaDu, Tu-139, 1483, HEp-2, HeLa, and RKO cells were cultured as previously described (Rey *et al*, 2000).

### Reverse transcription (RT)–PCR

Total RNA was isolated from the cultured cells using Trizol™ reagent (Invitrogen, Carlsbad, CA, USA) and was subjected to RNase-free DNase I digestion at 37°C for 2 h to eliminate any contaminating genomic DNA. Five *μ*g of DNA-free total RNA was dissolved in 15 *μ*l H_2_O, and RT reaction was performed in the first strand buffer (Invitrogen) containing 300 U Superscript II (Invitrogen), 10 mM dithiotrietol, 0.5 *μ*g random hexamer (Promega, Madison, WI, USA), and 125 *μ*M dNTP. The annealing reaction was carried out for 5 min at 65°C, and cDNA synthesis was performed for 2 h at 37°C, followed by incubation for 15 min at 70°C to stop the enzyme reaction. The RT product was diluted with 70 *μ*l H_2_O.

To amplify Bmi-1 cDNA, PCR reaction was performed with 2 *μ*l RT product using the following primers: 5′-AGCAGAAATGCATCGAACAA-3′ (forward) and 5′-CCTAACCAG ATGAAGTTGCTGA-3′ (reverse). The PCR amplification was allowed for 30 cycles at 94°C (1 min)/53°C (1 min)/72°C (1 min), followed by 7 min incubation at 72°C. This PCR condition with 1 *μ*l RT product allowed for exponential amplification of the starting cDNA. To control for the contamination with genomic DNA, we also performed PCR reactions using the samples without prior RT reaction. No visible amplification was obtained without RT. Polymerase chain reaction amplifications of *GPR1*, *Cyc2A*, *MMP-1*, *MMP-3*, and glyceraldehyde-3-phosphate dehydrogenase (*GAPDH*) were performed as described previously (Kang *et al*, 2003).

### Western blotting

Whole-cell extracts (WCE) were fractionated by SDS–polyacrylamide gel electrophoresis (PAGE) and transferred to Immobilon protein membrane (Millipore, Billerica, MA, USA). The antibodies for p16^INK4A^ (Ab-1) were purchased from Calbiochem (San Diego, CA, USA), and those for Bmi-1 (H99), *β*-actin (H-196), and CDK4 (C22) were from Santa Cruz Biotechnology (Santa Cruz, CA, USA).

### Bmi-1 gene amplification study

Genomic DNA was isolated from five strains of NHOK, two normal human oral fibroblasts (NHOF), and eight cancer cell lines, including six OSCC cells. The DNAs were digested with *EcoR*I and *Hind*III to completion, electrophoresed in 0.8% agarose gel, and transferred to nylon membrane. *Bmi-1* genomic sequences were probed by Southern hybridisation using radiolabelled probe synthesised from Bmi-1 cDNA or GAPDH cDNA, according to the methods described elsewhere (Kang *et al*, 1998). The Bmi-1 radioactive signal was detected by PhosphorImager and quantitated against that of GAPDH. The normalised quantitated values were used for statistical test (one way ANOVA) to compare the mean Bmi-1 radioactive signals in the NHOK cultures and in the OSCC cell lines.

### Immunohistochemical staining of Bmi-1 in paraffin-embedded tissues

*In situ* Bmi-1 expression was determined in oral mucosal tissue samples with normal (*n*=8), dysplastic (*n*=9), or cancerous (*n*=10) histopathology obtained from the Oral pathology diagnostic laboratoryat the University of California at Los Angeles School of Dentistry. The specimens were collected and processed according to the guidelines of the University of California at Los Angeles institutional review board. Immunohistochemistry was carried out with anti-Bmi-1 antibody (H-99, Santa Cruz, CA, USA) on 4-*μ*m-thick sections according to the methods described elsewhere (Kim *et al*, 2004a). The samples were counterstained with haematoxylin. Bmi-1 staining intensity per each sample was arbitrarily scored by an oral pathologist as negative (–), barely detectable (+/−), weak (+), moderate (++), or strong (+++; see 2). *In situ* Bmi-1 staining was also performed in cultured NHOK and SCC4 cells according to the previously described method (Kang *et al*, 2000).

### *In situ* staining for cellular senescence

Normal human oral keratinocytes cultures infected with the lentiviral vectors (LV-GFP and LV-Bmi-1i) were fixed in 2% formaldehyde/0.2%. glutaraldehyde for 3–5 min at room temperature. The cells were then stained for senescence-associated *β*-galactosidase (SA *β*-Gal) activity in freshly prepared staining solution (Dimri *et al*, 1995). The presence of SA *β*-Gal activity was evidenced by a dark-green colour in the perinuclear cytoplasmic region.

### Lentiviral vector construction and use of short-hairpin RNA (shRNA)

We utilised the lentivirus-based shRNA expression plasmid pLL3.7 to knockdown the expression of endogenous Bmi-1. The detailed method of using pLL3.7 to construct the lentivirus expressing shRNA is described elsewhere (Rubinson *et al*, 2003). We constructed pLL3.7-Bmi-1i using double-stranded oligonucleotide cassette containing the Bmi-1 target sequence (5′-AAGGAATGGTCCACTTCCATT-3′) as described previously (Bracken *et al*, 2003). The lentiviral vectors, LV-GFP and LV-Bmi-1i were prepared by transfecting 293 T cells with the RNA interference (RNAi) plasmids, respectively, pLL3.7 (insertless plasmid) and pLL3.7-Bmi-1i, using calcium phosphate transfection method in the presence of the packaging plasmid pCMVΔR8.2Vprx, and the envelope plasmid pCMV-VSV-G (Naldini *et al*, 1996). We constructed a lentivirus vector targeting the expression of *hTERT* (LV-hTERTi) using the plasmid pLL3.7-hTERTi, which expresses the shRNA containing the hTERT target sequence (5′-GGCCGATTGTGAACATGGA-3′; nucleotides 1947–1965 of the hTERT mRNA sequence, see GenBank Accession NM 198255). As a negative control, we constructed another lentiviral vector (LV-Cont.), which expresses nonfunctional shRNA containing the hTERT mRNA sequences (5′- GAACGTGCTGGCCTTCGGC-3′; nucleotides 337–357) that fails to inhibit the endogenous expression of hTERT. The viral supernatant was collected at 24–36 h after transfection and concentrated by ultracentrifugation, as previously described (Stewart *et al*, 1999). Ultracentrifugation led to the concentration of the original viral supernatant by at least 12.5 fold. Rapidly proliferating NHOK, SCC4, and BaP-T cells were infected with 1 ml of the concentrated virus (LV-GFP, LV-Cont., LV-Bmi-1i, and/or LV-hTERTi) in the presence of 6 *μ*g ml^−1^ polybrene for 3 h. This infection scheme invariably resulted in >90% infection efficiency determined by the percentage cells labelled with green fluorescence. The infected cells were photographed using an inverted epifluorescence microscope (Nikon, Melvill, NJ, USA).

## RESULTS

### Bmi-1 is overexpressed in OSCC cells and tissues

**Figure 1:**
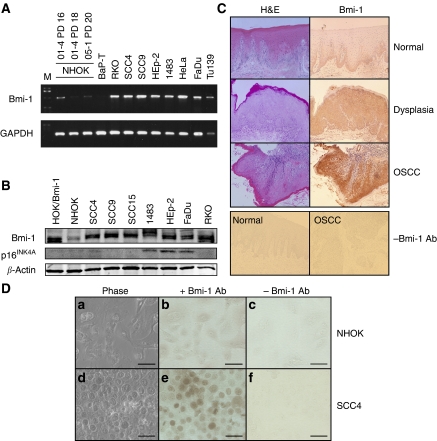
Bmi-1 is overexpressed in the cancer cells derived from OSCC. (**A**) Endogenous Bmi-1 expression level was compared by semi-quantitative RT–PCR in NHOK (01–4 and 05–1) and nine cancer cell lines including seven OSCC cell lines. Glyceraldehyde-3-phosphate dehydrogenase was amplified as a loading control. (**B**) Western blotting was performed to compare the Bmi-1 protein expression levels in NHOK and OSCC cells. HOK/Bmi-1, overexpressing exogenous Bmi-1 (Kim *et al*, 2006), was included as a positive control for Bmi-1 expression. In the same samples, we probed for the level of p16^INK4A^ to determine whether aberrant Bmi-1 expression correlated with downregulation of p16^INK4A^. *β*-actin was used as a loading control. (**C**) *In situ* immunohistochemistry was performed with oral mucosal tissue specimens showing the histological features of normal (*n*=8), dysplasia (*n*=9), and OSCC (*n*=10). The representative examples of histology (left panel, haematoxylin–eosin staining) and the Bmi-1 staining (right panel) are shown in this figure. Bmi-1 expression was detected by DAB staining (brown color). As negative controls, we included normal and OSCC specimens subjected to the staining procedure in the absence of specific Bmi-1 antibody (-Bmi-1 Ab). The images were captured with original magnification of × 100. (**D**) NHOK (a–c) and SCC4 (d–f) cells were fixed, permeabilised, and stained for Bmi-1 by indirect immunoperoxidase method. Bar=100 *μ*m.

To determine the association between Bmi-1 overexpression and oral carcinogenesis, we compared the expression levels of Bmi-1 in two independent cultures of NHOK and seven OSCC cells by semi-quantitative RT–PCR (Figure 1A and Table 1). RKO and HeLa cells, representing the colorectal and cervical cancer cells, respectively, were also included for comparison. All tested cancer cell lines expressed significantly higher level of Bmi-1 compared with those of NHOK. Replicating NHOK (strain 01-4, population doubling (PD) 16) expressed higher level of Bmi-1 than the senescing cells (strain 01-4, PD 18 and 05-1, PD 20). The Bmi-1 expression level was also determined by Western blotting in NHOK and the OSCC cell lines (Figure 1B). Bmi-1 protein was weakly detected in NHOK cultured in serum-free KGM and was not altered by the culture conditions (data not shown). As Bmi-1 is known to negatively regulate the expression of p16^INK4A^ in some cells (Jacobs *et al*, 1999), we checked for the correlation between the expression levels of Bmi-1 and p16^INK4A^ in our experimental system. Bmi-1 protein expression level was notably higher in most of the OSCC cell lines compared with that of NHOK, but did not correlate with the expression level of p16^INK4A^, which correlated more closely with the human papillomavirus (HPV) infection status (Table 1).

Bmi-1 protein expression *in situ* was determined by immunohistochemical staining of paraffin-embedded oral mucosal tissues with varying degrees of histopathology that covers the entire spectrum of oral carcinogenesis (Figure 1C). Bmi-1 protein expression, revealed by brown 3,3′-diaminobenzidine hydrochloride (DAB) staining, was weakly detectable and limited to the basal cell layer of normal stratified epithelium (*n*=8) and was not found in the upper spinosum and corneum layers. In contrast, all preneoplastic oral epithelial tissues (*n*=9) displaying mild, moderate, and severe dysplasia as well as malignant oral lesions (*n*=10) showed elevated Bmi-1 staining compared with those of the normal tissues (Table 2). The Bmi-1 staining in these aberrant tissues was detected in most of the epithelial layers containing viable cells, including stratum basale, spinosum, and granulosum. Stratum corneum and lucidum did not show Bmi-1 staining owing to the lack of viable cells in these layers. We also compared the levels of Bmi-1 staining *in situ* in cultured normal and cancer cells. Normal human oral keratinocytes and SCC4 were subjected to indirect immunopreoxidase staining for Bmi-1 using anti-Bmi-1 antibody. Diffuse cytoplasmic Bmi-1 staining was noted in NHOK, whereas SCC4 cells showed strong intranuclear and faint cytoplasmic Bmi-1 staining (Figure 1D). These data are in keeping with the Western blotting result, which showed notable increase in the Bmi-1 expression level in SCC4 cells compared with that of NHOK. The above results indicate that the elevated Bmi-1 expression is associated with preneoplastic oral lesions and is sustained in oral cancer.

### Bmi-1 gene is not amplified in the OSCC cell lines

**Figure 2:**
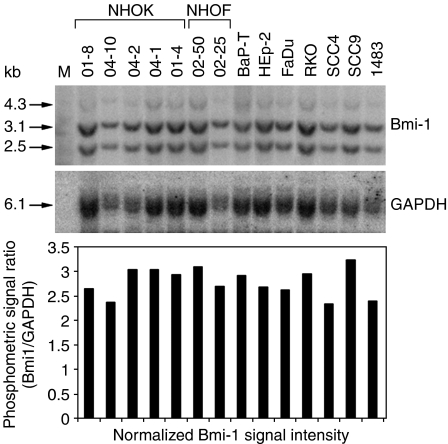
Bmi-1 gene is not amplified in OSCC. Genomic DNAs from five different NHOK strains, two NHOF strains, and seven cancer cell lines were digested with *EcoR* I and *Hind* III and transferred for probing. Radiolabelled probes synthesised from Bmi-1 or GAPDH cDNA were hybridised sequentially onto the membrane. The phosphometric intensities were plotted as the ratio of Bmi-1 to GAPDH. The lack of statistical difference (*P*>0.05) in the levels of Bmi-1 radioactive signals was determined by unpaired *T*-test (one-ways ANOVA) between the NHOK and the cancer groups.

To determine whether the Bmi-1 overexpression in the OSCC cells resulted from gene amplification, we compared the amount of Bmi-1 genomic sequences by Southern blotting in normal and cancer cells. We included five strains of NHOK, two NHOF strains, and seven cancer cell lines including six OSCC cell lines. The *Bmi-1*-specific signals were obtained at 4.3 kb, 3.1 kb, and 2.4 kb fragments, and were compared among the tested samples as its ratio to that of GAPDH (Figure 2). We found that there was no statistically significant difference in the relative abundance of the *Bmi-1* genomic sequences between the normal and the cancer cells. The Bmi-1 overexpression in OSCC may result from the mechanisms not involving gene amplification, such as promoter activation.

### Bmi-1 knockdown inhibits cellular proliferation of normal and cancer cells

**Figure 3:**
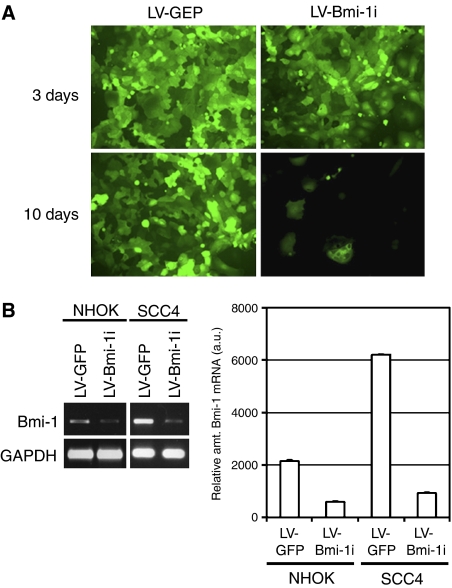
Inhibition of endogenous Bmi-1 causes replication arrest in SCC4 cells. (**A**) Rapidly proliferating SCC4 cells were infected with LV-GFP or LV-Bmi-1i. After 3 or 10 days, the images were obtained for GFP revealing those cells that were originally infected by the viral vectors. Original magnification, × 100. (**B**) Three days after infection of NHOK (05-10) and SCC4 cells with LV-GFP or LV-Bmi-1i, the level of Bmi-1 transcript was measured by semi-quantitative RT–PCR. The extent of Bmi-1 knockdown by LV-Bmi-1i was quantitated by Scion Image software against those of GAPDH amplification.

To investigate the biological role of Bmi-1, we knocked down the expression of endogenous Bmi-1 by RNAi in SCC4 cells and exponentially replicating NHOK. For this purpose, we constructed a lentiviral vector (LV-Bmi-1i) capable of expressing shRNA targeting Bmi-1 using the pLL3.7 plasmid, which also contains the green fluorescent protein (GFP) expression cassette under a heterologous promoter (Rubinson *et al*, 2003). SCC4 cells and replicating NHOK (strain 05–10, PD 10) were infected with LV-Bmi-1i and the control lentiviral vector (LV-GFP) expressing GFP alone. At 3 days post-infection, more than 95% of the cultures infected with LV-GFP or LV-Bmi-1i demonstrated green fluorescence (Figure 3A), indicating efficient infectivity. Also, at 3 days post-infection, the endogenous Bmi-1 expression level was decreased by 83 and 85%, respectively, in NHOK and SCC4 cells infected with LV-Bmi-1i if compared with the cells infected with LV-GFP (Figure 3B). The SCC4 cells were maintained in culture for longer than 10 days post-infection, during which time the cells infected with LV-Bmi-1i showed a marked reduction in proliferative capacity (Figure 3A). The LV-GFP-infected SCC4 cells continued to replicate beyond the 10-day period, whereas the SCC4 culture exposed to LV-Bmi-1i contained sparse, flattened cells with GFP expression.

**Figure 4:**
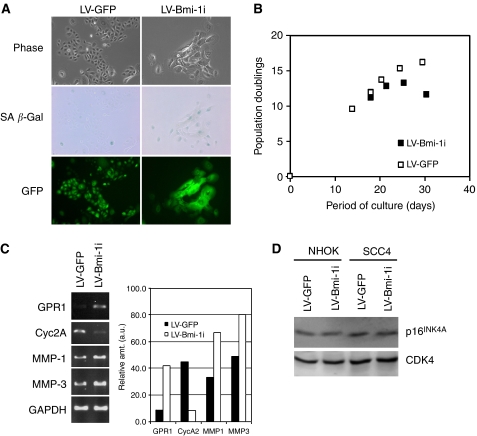
Inhibition of endogenous Bmi-1 causes premature senescence in NHOK. (**A**) Rapidly proliferating NHOK (05-10) was infected with LV-GFP or LV-Bmi-1i. Phase contrast photographs, SA *β*-Gal staining, and GFP fluorescence were obtained 10 days after virus infection. Original magnification, × 100. (**B**) Proliferation kinetics of NHOK infected with LV-GFP or LV-Bmi-1i was determined and plotted against time in culture. (**C**) NHOK cultures were harvested at 10 days after infection with LV-GFP or LV-Bmi-1i, and the expression levels of GPR1, Cyc2A, MMP-1, and MMP-3 were determined by semi-quantitative RT–PCR. The band intensities were quantitated and plotted by Scion Image software against those of GAPDH amplification. (**D**) NHOK (05-10) and SCC4 cells infected with LV-GFP or LV-Bmi-1i were harvested at 10 days after infection, and Western blotting was performed with 100 *μ*g WCE for p16^INK4A^. CDK4 was detected as a loading control.

At 3 days post-infection, the majority of NHOK also demonstrated the fluorescence signal (Figure 4A). Normal human oral keratinocytes infected with LV-GFP underwent 16 PDs before the onset of senescence, whereas those infected with LV-Bmi-1i expressed increased SA *β*-Gal activity and showed notable retardation of proliferation following the infection, completing only 12 cumulative PDs (Figure 4B). To determine whether the retarded proliferation in NHOK infected with LV-Bmi-1i represented the cellular senescence response, we compared the expression levels of *GPR1*, *MMP-1*, and *MMP-3*, which are the molecular markers of keratinocyte senescence (Kang *et al*, 2003). Semi-quantitative RT–PCR was performed with NHOK after 10 days post-infection (Figure 4C). Expression levels of GPR1, MMP-1, and MMP-3 were induced in NHOK infected with LV-Bmi-1i compared with those infected with LV-GFP. Also, the cells with Bmi-1 knockdown showed diminution of *Cyc2A* mRNA, of which the expression level is associated with exponential replication of NHOK (Kang *et al*, 2003). However, the cells infected with LV-Bmi-1i did not exhibit elevation of p16^INK4A^ (Figure 4D). These results demonstrated that the loss of endogenous Bmi-1 expression led to diminution of the cell proliferation capacity in both SCC4 cells and NHOK, in the absence of induced p16^INK4A^ expression level.

**Figure 5:**
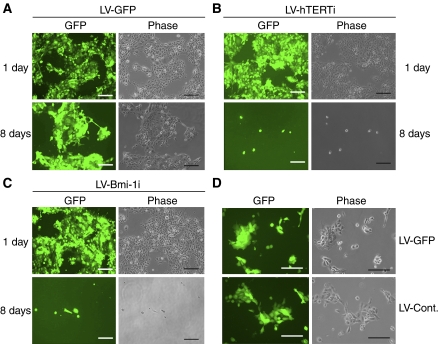
Inhibition of endogenous Bmi-1 led to effective loss of viability in BaP-T cells. Rapidly proliferating BaP-T cells were infected with LV-GFP (**A**), LV-hTERTi (**B**), LV-Bmi-1i (**C**), or LV-Cont (D). (**A**–**C**) The infected cells were labelled with green fluorescence owing to the GFP expression from the pLL3.7 parental lentiviral vector and shown along with the phase contrast view after 1 day or 8 days post-infection. The cells infected with LV-GFP were passaged owing to reaching confluence after 3–4 days, whereas those infected with LV-Bmi-1i or LV-hTERTi were maintained in the same dish without passaging for the period of observation. (**D**) In another experiment, BaP-T cells were infected with LV-GFP or LV-Cont. and maintained in parallel for 9 days and photographed. In this experiment, no notable differences in cellular morphology, replication capacity, or viability were noted between the two groups. Bar=200 *μ*m.

**Figure 6:**
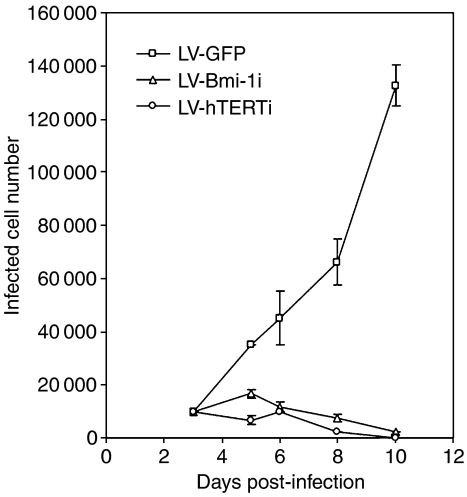
Effective loss of viability in BaP-T cells infected with LV-Bmi-1i or LV-hTERTi. Viable BaP-T cells infected with LV-GFP, LV-Bmi-1, or LV-hTERTi shown in Figure 5 were quantitated and plotted against period of culture. The total numbers of fluorescence-labelled adherent cells were determined at 3, 5, 6, 8, and 10 days post-infection.

The involvement of the p16^INK4A^ in the cell proliferation arrest triggered by Bmi-1 knockdown was further analysed by utilising the BaP-T cells, which possess aberrant p16^INK4A^/pRb pathway owing to the expression of HPV oncoprotein E7 (Park *et al*, 1995). Actively proliferating BaP-T cells were infected with LV-GFP or LV-Bmi-1i vectors. For comparison, we also infected the cells with LV-hTERTi, targeting the cellular telomerase activity, which demonstrated effective anticancer activity (Li *et al*, 2004). The cells were maintained in culture for 10 days post-infection, and the total numbers of the infected cells were determined. All of the lentiviral vectors demonstrated highly efficient infectivity even after 1 day post-infection (Figure 5). However, infection of BaP-T cells with LV-Bmi-1i or LV-hTERTi effectively inhibited cellular proliferation and led to loss of viability as early as 4 days post-infection (Figure 6). BaP-T cells were also infected with LV-Cont. expressing nonfunctional shRNA against hTERT sequence. These cells exhibited no phenotypic alteration compared with those infected with LV-GFP (Figure 5D). These results suggest that the intact p16^INK4A^/pRb checkpoint pathway is not necessary for the Bmi-1i-induced OSCC replication arrest and death.

## DISCUSSION

In the present study, we examined the possible involvement of the PcG protein Bmi-1 in oral carcinogenesis by (1) comparison of the Bmi-1 protein and RNA expression levels in normal and OSCC cells and tissues and (2) knockdown of endogenous Bmi-1 in normal and OSCC cells. The first part of the study revealed that Bmi-1 overexpression was consistently observed in the OSCC cells and tissues compared with the normal controls. The level of Bmi-1 expression in the OSCC cells was comparable to that of the cancer cells, that is, RKO and HeLa, derived from other cancer types. Importantly, Bmi-1 overexpression was also observed in 100% (9/9) of the preneoplastic oral mucosal tissues which included those with mild, moderate, or severe epithelial dysplasia. This finding indicates that Bmi-1 overexpression occurs very early during oral carcinogenesis and may be used as a biomarker of preneoplastic oral lesions. A recent study examined the expression status of Bmi-1 in the lung SCC precursor lesions, which showed Bmi-1 expression in 48% of the cases by immunofluorescent staining (Breuer *et al*, 2005). Thus, Bmi-1 overexpression and the development of preneoplastic lesions are more closely associated in the oral mucosa than in the bronchial tissues.

The mechanism leading to the enhanced Bmi-1 expression in OSCC cells is not clear. However, we ruled out the possibility of gene amplification by Southern blotting, which showed comparable level of Bmi-1 genomic sequences in normal and OSCC cells. Using the Bmi-1 promoter-luciferase construct, we found that the promoter activity was strongly induced in the OSCC cells compared with that of NHOK (unpublished observation), indicating that Bmi-1 overexpression results from promoter activation. Recent studies showed that Bmi-1 is a direct transcriptional target of c-Myc in human fibroblasts (Guney *et al*, 2006) and of E2F-1 in neuroblastomas (Nowak *et al*, 2006). *c-Myc* was found to be frequently amplified in OSCCs by comparative genomic hybridisation (Chen *et al*, 2004). Thus, *c-Myc*-dependent Bmi-1 promoter activation may account for the enhanced gene expression during dysplastic cell transformation in oral carcinogenesis, although the extent to which c-Myc regulates the *Bmi-1* promoter activity in oral carcinogenesis needs further investigation.

Bmi-1 knockdown led to inhibition of cellular replication in both NHOK and OSCC cells. However, the cellular response seems to be different between the normal and cancer cells. In NHOK, Bmi-1 knockdown did not elicit an immediate response of replication arrest and/or loss of viable cells, although the cellular replication rate was notably reduced and the senescence prematurely triggered. In contrast, SCC4 and BaP-T cells underwent rapid arrest of replication upon infection with LV-Bmi-1i and showed drastic decrease in the viable cell number. After 8–10 days post-infection, the majority of the OSCC cells detached from the culture dish (Figures 3A and 5). Comparison of the replication kinetics of the cells infected with the LV-GFP or LV-Bmi-1i also demonstrates clear difference in the cellular response between NHOK and the BaP-T cells (Figures 4B and 6). Our result is consistent with a recent report demonstrating cancer-specific cytotoxic effect of Bmi-1 knockdown in neuroblastoma cells (Liu *et al*, 2006). The differential effect of Bmi-1 knockdown in the normal and cancer cells may be beneficial if Bmi-1 were to be targeted for anticancer therapy.

The LV-Bmi-1i vector was effective against the BaP-T cells, which express HPV type 16 E7 (Park *et al*, 1995). Thus, Bmi-1i-mediated growth inhibition may not require intact p16^INK4A^/pRb checkpoint pathway, as it is also reflected in the absence of p16^INK4A^ induction in the NHOK and SCC4 cells after Bmi-1 knockdown. The inverse correlation between Bmi-1 and p16^INK4A^ protein expression levels was not evident in the OSCC cell lines in our study (Figure 1B). Furthermore, we recently reported that expression of exogenous Bmi-1 in NHOK led to significant extension (2.7 fold) of replicative life span but did not efficiently downregulate the expression of p16^INK4A^ (Kim *et al*, 2006). It is possible that Bmi-1 targets other important regulators of cell division in NHOK, such as p14^ARF^ or p15^INK4B^, as previously suggested (Jacobs *et al*, 1999). Several recent studies also showed that the aberrant Bmi-1 expression is not necessarily associated with downregulation of p16^INK4A^ expression (Dukers *et al*, 2004; Breuer *et al*, 2005). Also, Bracken *et al* (2006) recently identified numerous cellular genes targeted by Bmi-1 other than p16^INK4A^ using microarray-based expression screening and chromatin immunoprecipitation (ChIP)-on-chip analysis. These findings support the possibility that Bmi-1 elicits its oncogenic property during oral carcinogenesis in p16^INK4A^-independent manner.

## Figures and Tables

**Table 1:** The OSCC cell lines and the status of Bmi-1, p16^INK4A^, p53, and HPV infection

**Cells**	**Tissue origin**	**Bmi-1^a^**	**p16^INK4A^^b^**	**p53**	**HPV status**
BaP-T^c^	gingiva	−	+	Targeted by E6	+/type 16
SCC4^d^	tongue	+	+	mutant	−
SCC9^d^	tongue	+	−	−	−
SCC15^d^	tongue	+	−	−	−
HEp-2^d^	larynx	+	+	Targeted by E6	+/type 18
1483^e^	oral	+	+	Targeted by E6	+/type 18
FaDu^d^	pharynx	+	+	Mutant	−
Tu139^f^	larynx	+	−	Mutant	−
RKO^g^	colorectum	+	−	wild-type	−
HeLa^d^	cervix	+	+	Targeted by E6	+/type 18

a Bmi-1 overexpression was determined based on the RT–PCR and Western blotting results (Figure 1). Positive indicates enhanced expression compared with the baseline (negative) expression level found in the NHOK cultures.

b p16^INK4A^ expression status is based on the Western blotting results (Figure 1B).

c Tumourigenic counterpart of NHOK (Park *et al*, 1995).

d Purchased from American Type Culture Collection. The protein expression patterns for these cell lines were based on Munro *et al*, 1999.

e Gift of P. Sacks (Univ. Texas, Houston, TX, USA).

f Gift of G. Clayman (Univ. Texas, Houston, TX, USA).

g Gift of M. Kastan (Johns Hopkins, Baltimore, MD, USA).

**Table 2:** Bmi-1 immunoreactivity *in situ* is elevated in the oral epithelium with dysplastic and cancerous histopathology

**Pathologic category**	**Specimen no.**	**Histopathologic finding**	**Bmi-1^a^**
Normal (*n*=8)^b^	1	Normal oral epithelium	+
	2	Normal oral epithelium	+/−
	3	Normal oral epithelium	+/−
	4	Normal oral epithelium	−
	5	Normal oral epithelium	−
	6	Normal oral epithelium	+/−
	7	Normal oral epithelium	+/−
	8	Normal oral epithelium	−
Dysplasia (*n*=9)^c^	1	Moderate-severe epithelial dysplasia	+++
	2	Focal keratosis and mild epithelial dysplasia	++
	3	Mild epithelial dysplasia	+
	4	Moderate-severe epithelial dysplasia	+++
	5	Moderate-severe epithelial dysplasia	++
	6	Hyperkeratosis and mild epithelial dysplasia	++
	7	Severe epithelial dysplasia	+++
	8	Mild epithelial dysplasia	++
	9	Severe epithelial dysplasia	+++
HNSCC (*n*=10)^c^	1	Moderately differentiated squamous cell carcinoma	++
	2	Moderately well differentiated squamous cell carcinoma	++
	3	Superficial moderately differentiated squamous cell carcinoma	+++
	4	Poorly differentiated squamous cell carcinoma	++
	5	Moderately differentiated squamous cell carcinoma	+
	6	Well-differentiated squamous cell carcinoma	+
	7	Well-differentiated squamous cell carcinoma	+
	8	Moderately differentiated squamous cell carcinoma	++
	9	Well-differentiated squamous cell carcinoma	+++
	10	Moderately differentiated squamous cell carcinoma	+++

a The level of Bmi-1 immunostaining per each specimen was scored as negative (−), barely detectable (+/−), weak (+), moderate (++), or strong (+++) by an oral pathologist, noting the level of chromogenic development after addition of the DAB substrate. The scoring was confirmed blindly by an individual without prior knowledge or understanding of the nature of the tissue specimens.

b Bmi-1 in normal oral epithelium was limited to the basal cell layer (Figure 1C).

c Bmi-1 staining in the dysplastic and the OSCC samples was homogenously detected in most of the epithelial layers including stratum basale, spinosum, and granulosum (Figure 1C).
